# Nanoscale Interaction of Endonuclease APE1 with DNA

**DOI:** 10.3390/ijms25105145

**Published:** 2024-05-09

**Authors:** Sridhar Vemulapalli, Mohtadin Hashemi, Yingling Chen, Suravi Pramanik, Kishor K. Bhakat, Yuri L. Lyubchenko

**Affiliations:** 1 Department of Pharmaceutical Sciences, University of Nebraska Medical Center, Omaha, NE 68198-6025, USA; sridhar.vemulapalli@unmc.edu (S.V.); hashemi@auburn.edu (M.H.); 2 Department of Physics, Auburn University, Auburn, AL 36849-5318, USA; 3 Department of Genetics, Cell Biology and Anatomy, University of Nebraska Medical Center, Omaha, NE 68198-5805, USA; yingling.chen@unmc.edu (Y.C.); spramanik@tesseratx.com (S.P.)

**Keywords:** apurinic/apyrimidinic endonuclease 1, APE1 endonuclease, G-quadruplexes, protein–DNA complexes, AFM imaging, DNA looping

## Abstract

Apurinic/apyrimidinic endonuclease 1 (APE1) is involved in DNA repair and transcriptional regulation mechanisms. This multifunctional activity of APE1 should be supported by specific structural properties of APE1 that have not yet been elucidated. Herein, we applied atomic force microscopy (AFM) to characterize the interactions of APE1 with DNA containing two well-separated G-rich segments. Complexes of APE1 with DNA containing G-rich segments were visualized, and analysis of the complexes revealed the affinity of APE1 to G-rich DNA sequences, and their yield was as high as 53%. Furthermore, APE1 is capable of binding two DNA segments leading to the formation of loops in the DNA–APE1 complexes. The analysis of looped APE1-DNA complexes revealed that APE1 can bridge G-rich segments of DNA. The yield of loops bridging two G-rich DNA segments was 41%. Analysis of protein size in various complexes was performed, and these data showed that loops are formed by APE1 monomer, suggesting that APE1 has two DNA binding sites. The data led us to a model for the interaction of APE1 with DNA and the search for the specific sites. The implication of these new APE1 properties in organizing DNA, by bringing two distant sites together, for facilitating the scanning for damage and coordinating repair and transcription is discussed.

## 1. Introduction

Apurinic/apyrimidinic endonuclease 1 (APE1) is a multifunctional protein involved in DNA repair, specifically base excision repair (BER) and nucleotide incision repair (NIR) [1,2,3,4]. APE1 is essential for maintaining genomic stability by repairing the DNA damage caused by reactive oxidation species, alkylating agents, and ionizing radiation [4,5,6]. APE1 cleaves abasic sites in the DNA, which occur due to damage or as a repair intermediate [1,2,5,7]. In addition to its role in DNA repair, APE1 interacts with transcription factors such as p53, NF-κB, and HIF-1α and plays a vital role in DNA replication and is involved in resolving stalled replication forks by repairing the damaged DNA during DNA replication, repair, and recombination processes [3,5,6,8,9,10,11]. It has been reported that APE1 interacts with several key enzymes such as DNA polymerase delta and stimulates the activity of flap endonuclease 1 (FEN1) [6,9,12,13].

APE1 interacts with and binds G4 structures formed by G-rich DNA sequences, suggesting its role in regulating gene expression [1,2,14,15,16]. In addition, APE1 has been reported to regulate the stability and dynamics of G4 structures by nicking or cleaving the DNA backbone at specific positions, which affects the folding and unfolding of the G4 structure [1,2,5,10]. APE1 interacts with other G4-binding proteins, such as nucleophosmin and hnRNPU, and regulates their binding to G4 structures [1,2,16,17,18]. APE1 interactions with G4 structures and binding proteins offer potential therapeutic targets for cancer treatment and other diseases [1,2,3,5,7,8,9,10,19,20,21,22].

Enrichment of APE1 in gene regulatory regions and participation in transcriptional regulation led to the hypothesis that APE1 can bring together two specific DNA segments on the same DNA molecules, forming a loop. DNA looping is a fundamental mechanism in many processes, in particular the transcriptional initiation in both prokaryotes and eukaryotes, and brings distant sites close to the promoter region [23,24,25,26,27]. The formation of DNA loops requires binding to two or more DNA segments, with loop formation being achieved by the interaction of a single protein with two or more sites or by binding two or more DNA segments through a multimer. However, there is no direct evidence of APE1-mediated DNA looping.

We address these questions using AFM to characterize the APE1–DNA complexes directly. We have previously shown that AFM is instrumental in imaging various protein–DNA complexes, reviewed in [28]. Specifically, we characterized looped protein–DNA complexes, such as those formed by restriction enzymes [29]. Importantly, using AFM, we identified additional DNA binding sites in EcoRII endonuclease, allowing the formation of double-looped complexes [30]. Herein, we applied AFM to characterize the interaction of APE1 with DNA using a DNA substrate containing two well-separated G-rich segments. Using this approach, we demonstrate the affinity of APE1 to G-specific motifs. The formation of loops was also demonstrated, but in addition to specific loops between the G-rich segments, non-specific loops are also formed. However, no G-quadruplex structures were identified on the DNA substrate alone, suggesting that their formation is not required for APE1-specific binding and that such structures can be stabilized by APE1 binding. Finally, loops are formed by the monomeric APE1 protein, suggesting that the protein has two DNA binding sites.

## 2. Results

### 2.1. DNA Design: Preparation of the Substrate with Two APE1 Sites

**Figure 1:**
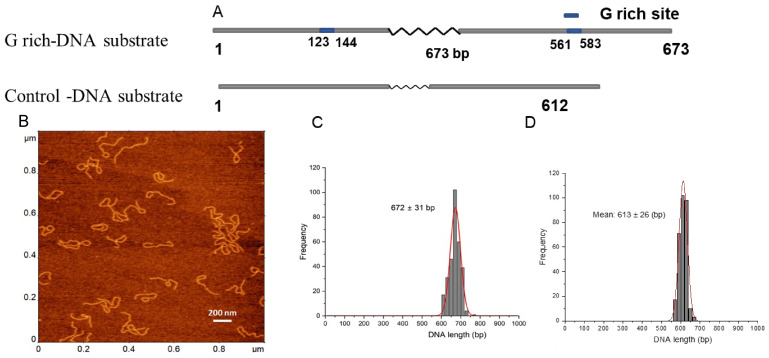
DNA substrates, AFM image, and contour length. (**A**) The schematic for the G rich-substrates (upper scheme) and the control (bottom). 22 bp G-rich motifs are located at 123–144 bp and at 561–583 bp and are shown in blue. The non-G-rich DNA substrate with 612 bp in length was used as a control. (**B**). A typical 1 × 1 μm AFM scan of G-rich DNA substrate (**C**,**D**) are histograms for the contour length measurements for G-rich DNA substrate and the control, respectively. Each distribution is approximated with single Gaussians built with a bin size of 20 bp. The contour length values in base pairs and standard deviations are indicated for each histogram.

We used a 673 bp DNA substrate from the human genome containing two 22 bp long G-rich motifs of the *c-MYC* gene regulatory region. G-rich segments were located at positions 123 bp and 583 bp of the DNA substrate (Figure 1A). The DNA was selected based on the previous biochemical studies conducted to characterize the APE1 interactions [1,2]. The two G-rich sites on the DNA are separated by 417 bp, which, according to our previous publications, is appropriate for the assembly and the AFM visualization of the protein-mediated DNA loops [29].

Typical AFM images of the G-rich DNA substrate are shown in Figure 1B, in which DNA appears as smooth filaments. The contour length measurements are shown in Figure 1C. A total of N = 300 particles was analyzed, and a single peak Gaussian function approximation of the histogram gives a mean of 672 ± 31 bp (SD). Similar contour length measurements of the control DNA of 612 bp with no G-rich segments are shown in Figure 1D.

### 2.2. APE1 Complex Assembly and Loop Size Analysis

**Figure 2:**
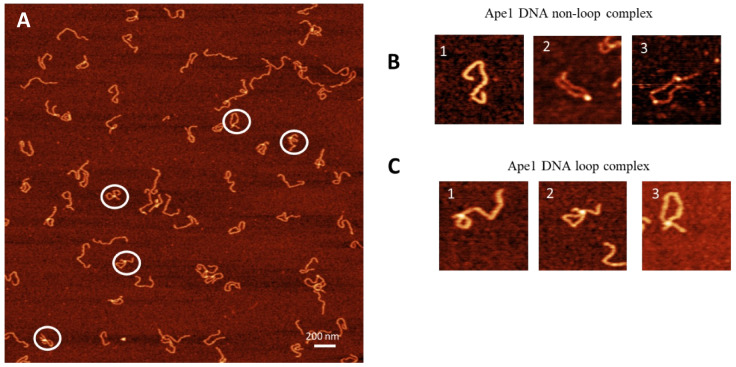
AFM image of complexes of APE1 with G-rich DNA complexes (1:1). (**A**) The AFM image with looped complexes of APE1-G-rich-DNA. Zoomed images of complexes circled in (**A**) are indicated in (**B**,**C**). (**B**) A set of images with no APE1 bound (frame 1) and non-looped complexes with one APE1 bound (frame 2) and two APE1 bound (frame 3). (**C**) A set of three looped complexes with different sizes of loops.

The APE1–DNA complexes were assembled at a 1:1 protein/DNA ratio and prepared for AFM imaging. AFM images of the APE1–DNA complexes are shown in Figure 2A with a few zoomed images shown in Figure 2B,C. Three different morphologies were identified: bare DNA (Figure 2B(1)); DNA with APE1 as bright globular features (Figure 2B(2,3)) and looped DNA with APE1 as globular features (Figure 2C(1–3)). The overall yield of complexes is 53%, with the partition of looped and non-looped complexes 22% and 31%, respectively (Table 1).

**Figure 3:**
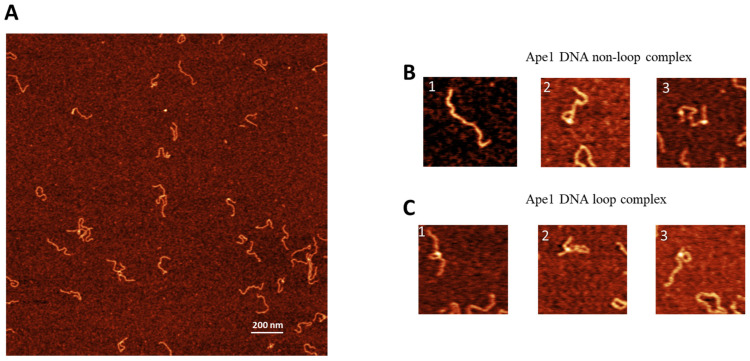
AFM image of complexes of APE1 with non-G-rich DNA complexes (control substrate). (**A**) A typical AFM scan with 3 × 3 in size. shows the AFM image with looped complexes of APE1–non-G rich-DNA. (**B**) and (**C**) show a few examples of complexes with linear morphology and looped DNA complexes, respectively.

Similar experiments were performed for the control DNA substrate containing no G-rich sequences. The AFM images are shown in Figure 3A. Similar to the G-rich DNA substrate, three different morphologies were observed with selected zoomed images shown in Figure 3B,C. These are free DNA (Figure 3B(1)), DNA with bright features (Figure 3B(2,3)), and looped complexes (Figure 3C(1–3)). The overall yield of complexes is 19%, with the yield of looped complexes being 4% (Table 1).

### 2.3. AFM Data Analysis: Positioning of APE1 on DNA

**Figure 4:**
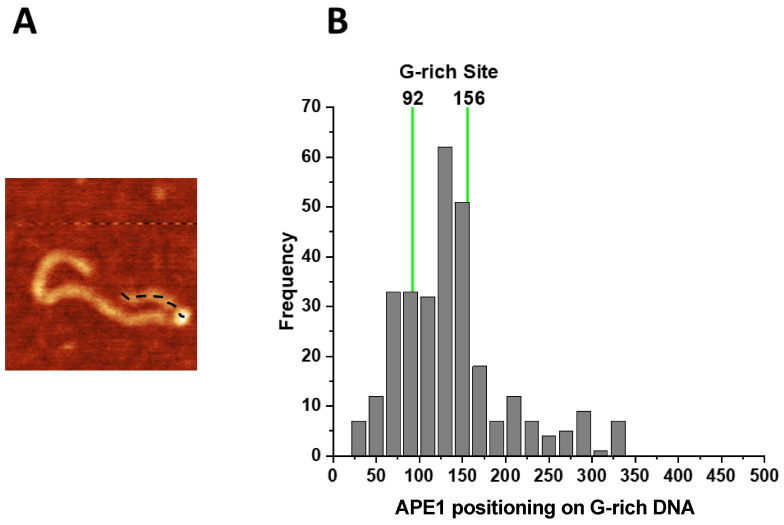
Mapping of the APE1 positions on the G-rich-DNA substrate. (**A**) AFM image of APE1–G rich-DNA complex. The dotted line illustrates the contour length of the short arm measured from the DNA end to the center of the protein. (**B**) The histogram of APE1 mapping performed over 300 molecules. Vertical green lines correspond to the range of distances from both DNA ends to G-rich motifs, which includes the 22 bp size of the motifs. Locations of APE1 within the 92–156 bp range correspond to the specific binding of the protein.

Given the relative symmetry in the position of G-rich segments on the DNA, relative to the DNA ends (123–144 bp and 561–583 bp), we mapped the positions of APE1 on the G-rich DNA by measuring the length of the distance between the bright features and closest DNA end (Figure 4A). The measurements were made for 300 complexes and the results are shown as a histogram in Figure 4B. Green vertical lines indicate positions of the G-rich motifs in the DNA molecule. Positions of APE1 within the range of the green lines are considered as specific interactions of APE1 with DNA.

A similar analysis was carried out for complexes of APE1 with the control DNA substrate. The histogram of the APE1 position measured from the end of the DNA molecule is shown in Figure S4A.

### 2.4. AFM Data Analysis: Sizes of DNA Loops

**Figure 5:**
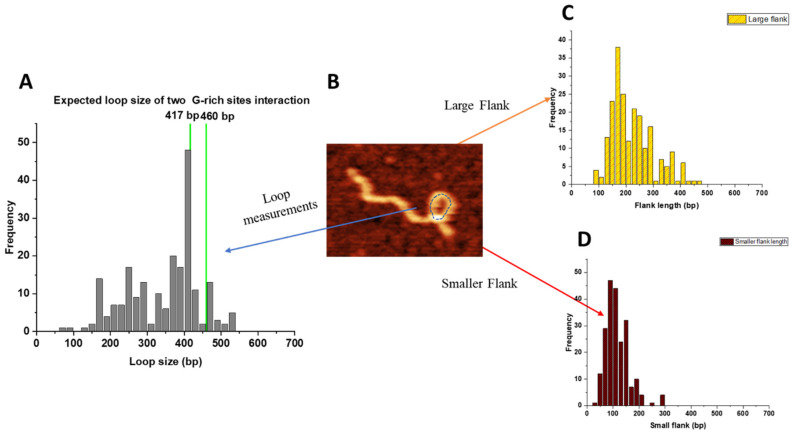
Looped complexes formed by APE1 on the G-rich DNA substrate. (**A**) The histogram for the loop sizes obtained for 200 looped complexes. Vertical green lines indicate the sizes of loops formed by bridging of two G-rich motifs, which includes their sizes. (**B**) AFM image showing the looped complex. The loop is indicated using a dotted line. (**C**) The histogram of the lengths of the long arms. (**D**) The histogram of the lengths of short arms.

For looped APE1–DNA complexes, two parameters were measured: the loop sizes and the lengths of the flanks. The results are assembled in Figure 5. The loop sizes are shown in Figure 5A and have a narrow distribution around 410 bp with a spread between ~350 bp and ~450 bp, which, when taking into account the 22 bp size of the G-rich motifs, corresponds to the assembly of complexes between the G-rich sites. Data beyond this size correspond to the formation of non-specific loops.

The results for measurements of short and long flanks are shown in Figure 5C,D, respectively. The short flank length distribution is narrow and spans over the range of 60–150 bp, which, due to the 22 bp length of the G-rich sites, covers the expected position for binding APE1 to one or the other G-rich sites. On the other hand, the distribution of the lengths of the long arm is broad. In addition to the range corresponding to APE1 binding to one or the other G-rich sites (vertical lines), events corresponding to the assembly of loops with APE1 binding to non-specific sites are also present.

The yield of looped complexes for the control DNA substrate was 4%, which is ~1/6 compared to complexes assembled on a G-rich DNA substrate (see Table 1). The control substrate’s loop sizes were analyzed, and the data are shown in Figure S4B. The distribution was broad and flat, with no preferential loop size identifiable, indicative of a random distribution, which is corroborated by simulated distribution for non-specific looping (Figure S5).

### 2.5. Looped Structures Are Formed by Monomeric APE1

AFM captures the 3D shape of molecules and allows evaluation of their sizes. We used the height and volume measurements to estimate the molecular weights of proteins complexed with DNA [31,32]. This information clarifies whether APE1 monomers, dimers, or larger oligomers are responsible for assembling loops. The two different pathways impose certain conditions; in the case of the dimeric stoichiometry in the looped complexes, each monomer should bind to DNA first, and then the loop is formed via protein–protein interactions. If the monomeric APE1 makes loops, the protein should have two DNA binding sites.

**Figure 6:**
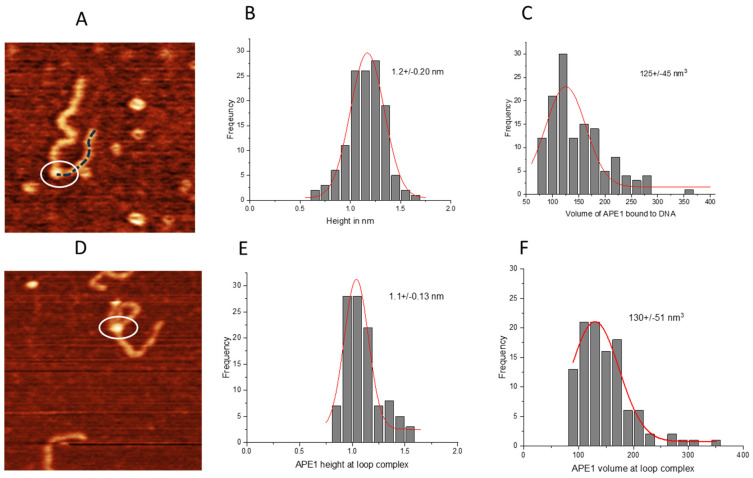
The height and volume analysis of the APE1 on G-rich DNA with the non-looped and looped complexes. (**A**) AFM image of the APE1 protein positioned on linear DNA. Circle highlights the APE1 protein, while dotted line illustrates the short DNA flank. (**B**) Histograms for height values of the APE1 protein approximated with a Gaussian distribution (1.2 ± 0.20 nm). (**C**) The histogram of the volume measurements data approximated with a Gaussian distribution (125 ± 45 nm^3^). (**D**) AFM image of looped complexes of APE1 protein (circled). (**E**) The histogram for the protein height approximated with a Gaussian distribution (1.1 ± 0.13 nm). (**F**) The histogram for the protein volume approximated with a Gaussian distribution (130 ± 51 nm^3^).

First, we measured the APE1 protein height in non-looped complexes with DNA on the G-rich substrate (Figure 6A) and obtained the height histogram (Figure 6B), which approximated with a Gaussian shows a peak at 1.2 ± 0.2 nm. The corresponding volume of the protein displayed a Gaussian distribution with a mean value of 125 ± 45 nm^3^ (Figure 6C). Next, we measured the same parameters for APE1 in looped complexes (Figure 6D). The height histogram for the protein in looped complexes was approximated with a Gaussian distribution and yielded a mean value of 1.1 ± 0.13 nm (Figure 6E). Similarly, the volume of the protein displayed a Gaussian distribution centered around 130 ± 51 nm^3^ (Figure 6F).

**Figure 7:**
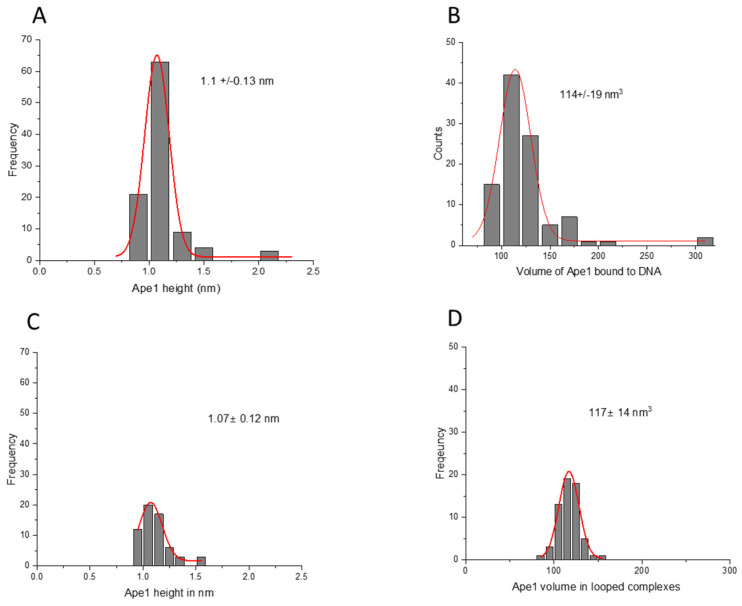
Height and volume measurements for complexes of the APE1 on control DNA substrate with non-looped and looped complexes. (**A**) and (**B**) are the histograms for the protein heights and volume, respectively, for non-looped complexes. (**C**) and (**D**) are the histograms for the height and volume of APE1, respectively. Each histogram is approximated by single Gaussians with parameters indicated in the plots.

As a control, we measured the height and volume of the APE1 protein in complexes with the control DNA substrate. The height of the protein in this complex showed a Gaussian distribution with a mean value of the height of 1.1 ± 0.13 nm, as shown in Figure 7A. The volume of the protein exhibited a Gaussian distribution centered around 114 ± 19 nm^3^ (Figure 7B). The height and volume of the APE1 protein in looped complexes with the control DNA produced values 1.07 ± 0.12 nm and 117 ± 14 nm^3^ (Figure 7C,D), which are indistinguishable from those obtained for the G-rich DNA substrate.

**Figure 8:**
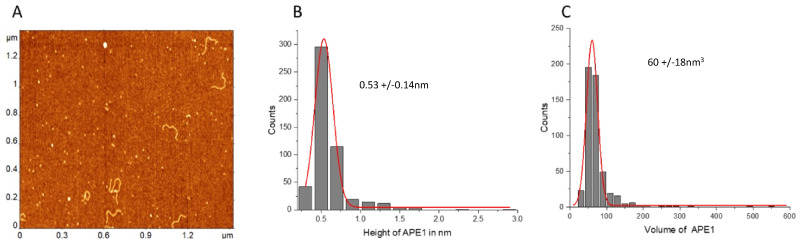
Height and volume measurements of the free APE1 protein. (**A**) AFM images of the free protein with added DNA as a reference. (**B**,**C**) are the histograms for the height and volume values built for 100 measurements. The histograms are approximated with Gaussians with parameters indicated in the plots.

Height measurements of free APE1 produced the value 0.53 ± 0.14 nm (Figure 8B), which, combined with the DNA height ~0.5 nm, produces the height value ~1.1 nm (Figure 8C) for the protein bound to DNA. This value is close to the height values measured for protein bound to DNA, suggesting that protein binding to DNA is not accompanied by its oligomerization.

These findings suggest that the monomeric form or single APE1 is involved in bridging two distant sites, suggesting that APE1 has two DNA binding segments and both are involved in the DNA looping.

## 3. Discussion

AFM studies clarified several novel features involved in the interaction of APE1 with DNA.

The binding of APE1 to the G-rich motifs was previously shown using various indirect studies; here, visualization with AFM directly evaluated the specificity of APE1 binding to G-rich segments. In addition, analysis of AFM data revealed that APE1 is capable of binding to non-G DNA as well, with the yield of such complexes being approximately two-fold lower than the formation of specific APE1–G complexes (Figure S4).

Studies have shown that quadruplex formation and stability can depend on the ionic species present [33,34,35,36]. Herein, experiments were performed in the presence of K^+^ ions, which have been shown to be favorable for the formation of quadruplex structures [35,36,37]. However, AFM images of only DNA (Figure 1) demonstrated that the G-rich DNA molecules are smooth and indistinguishable from the control, without G-rich segments, in contrast to G quadruplexes routinely visualized with AFM [38,39]. Note that in vitro colocalization studies showed APE1 localization to quadruplex sites [1,2]. These studies lead the authors to hypothesize that, in cells, it is not the G-rich dsDNA sequence per se but the formation of quadruplex DNA secondary structure that recruits APE1 to the promoter–enhancer regions to regulate repair and transcription. However, studies in this paper demonstrate that no quadruplexes are formed stably in the G-rich DNA substrate. At the same time, it has been reported that APE1 stabilizes quadruplex structures [1]. It is not possible to detect DNA morphology with APE1 bound to the G-rich segment, as the protein will cover the bound DNA segment. However, a change in DNA morphology should translate to a change in protein–DNA complex height and volume. Neither height nor volume of APE1 (Figure 6 and Figure 7), bound to DNA or participating in loop formation, were significantly different when comparing APE1 interacting with the G-rich DNA construct versus the control DNA construct without G-rich segments.

Looping was another putative function of APE1 that we provide evidence for here. APE1 is capable of binding two sites on the same DNA molecule, leading to the formation of looped DNA structures. Analysis of AFM data showed that loops of different sizes are formed, and loops corresponding to the bridging of two G-rich segments were also visualized. The yield of such G-specific loops is close to the yield of non-specific loops, which is in line with the findings regarding the binding of APE1 to G-rich and non-specific linear DNA. However, additional quantitative analysis of looped complexes revealed an interesting assembly feature. As demonstrated in Figure 5, looped complexes in the vast majority of cases have short flanks with the length corresponding to the position of G-rich sites. In other words, APE1 in the loops binds to one G-rich site and one other site, which can be another G-rich site or any non-G-rich segment.

The AFM images allowed us to elucidate the APE1 stoichiometry in the looped complexes. We determined the stoichiometry of APE1 in looped complexes by performing measurements of the protein sizes. The data shown in Figure 2, Figure 3 and Figure 4 demonstrate that looped complexes are formed by monomeric APE1 rather than its dimer. APE1 multimers were expected based on previous studies by Kladova et al., which show that APE1 multimers are integral for their function in the base excision repair process [40]. Bridging of two DNA binding sites is possible if the proteins have multiple DNA binding sites [30]. The binding of two DNA segments by the monomeric APE1 suggests the protein has two binding sites. As we discussed above, looped complexes on the G-rich DNA substrate almost always have APE1 bound to one G-rich segment. This finding leads to the hypothesis that one DNA binding site of APE1 has a strong specificity to G-rich sequences, and the other site is more promiscuous.

We recently proposed the model for the site search process during DNA looping based on studies of the highly sequence-specific restriction enzyme SfiI [29]. According to this model, during the search process, the protein initially binds to a specific site, grabs any non-specific site, and threads DNA in search of another specific site. In the framework of this model, we hypothesize that APE1 binds to the G-rich region on DNA at its specific site and searches DNA by using its less specific site. Note that such a mechanism has recently been proposed for the DNA looping for cohesin [41]. APE1-mediated DNA looping for bringing two distant sites together may facilitate damage search in the transcriptional regulatory regions, coordinating repair and long-range promoter–enhancer interaction for repair and transcription.

## 4. Materials and Methods

### 4.1. APE1-Protein

The full-length APE1 coding sequence was inserted in the pET15b vector (Novagen, Madison, WI, USA) at NdeI/Xho I sites for expression of APE1 in the *E. coli* Rosetta 2 strain (Novagen). The DNA sequence of the APE1 was confirmed by the UNMC genomic core. APE1 protein was purified as previously described [42] with slight modifications. After transforming with the pET15b-based APE1 expression plasmid, *E. coli* were grown to 0.6 OD at 600 nm. APE1 expression was then induced with 0.5 mM isopropyl-β-D-thiogalactopyranoside (IPTG) at 18 °C for 16 h. The cells were then suspended in a buffer containing 20 mM Tris (pH 8.0) and 0.5 M NaCl, sonicated, and centrifuged. The supernatant was loaded onto the Ni-NTA (Qiagen, Germantown, MD, USA) column (3 mL), run, and then eluted with buffer containing 200 mM imidazole. The eluate was dialyzed against 20 mM Tris-Cl (pH 8.0), 100 mM NaCl, 1 mM EDTA, 1 mM dithiothreitol (DTT), and 10% glycerol. The poly His-tag in the protein was cleaved by overnight incubation at 4 °C with thrombin. The APE1 was finally purified by FPLC using an SP-Sepharose column (LCC-500 PLUS; Pharmacia, Chicago, IL, USA), and the final preparation was dialyzed against 20 mM Tris (pH 8.0), 300 mM NaCl, 0.1 mM EDTA, 1 mM DTT, 50% glycerol, and stored at −20 °C.

### 4.2. DNA Substrates

A 673 bp DNA segment of the *c-MYC* gene promoter (−25 to −648 bp with respect to the transcription start site) was amplified by PCR and formed the DNA substrate containing two G-rich motifs (Figure 1A). For the PCR reaction, 100 ng of human genome DNA and PfuUltra High-Fidelity DNA polymerase (#600380) were used with the primers: Forward primer: AGGGTTTGAGAGGGAGCAAAAG; Reverse primer: CTCGGGTGTTGTAAGTTCCAG. Similarly, DNA without G-rich motifs with a length of 612 bp, as shown in (Figure 1A), was obtained by performing PCR of the plasmid.

Both DNA substrates were gel-purified as described [29]. Briefly, the PCR product was run on a 1% agarose gel. The product bands corresponding to the expected length of the DNA were excised, and DNA was extracted and purified using the Qiagen DNA gel extraction kit (Qiagen Inc., Valencia, CA, USA). The final DNA concentration was determined by absorbance at 260 nm using a NanoDrop spectrophotometer (NanoDrop Technologies, Wilmington, DE, USA).

### 4.3. APE1-DNA Synaptosome Assembly

DNA was mixed with APE1 enzyme at the molar ratio 1:1 in 50 mM Tris-HCl buffer containing 50 mM KCl, 2 mM MgCl_2_ with a total volume of 10 μL, and with the final concentrations of DNA and APE1 at 1 nM. A reaction mixture for APE1-DNA assembly consisted of a final volume of 10 μL with 7 μL of 1X buffer A [50 mM Tris HCl (pH 7.5), 50 mM KCl, 2 mM MgCl_2_, 0.1 mM EDTA], 1 μL of 10 mM DTT, 1 μL of DNA, and 1 μL of protein. The reaction mixture was incubated for 15 min at room temperature.

### 4.4. Atomic Force Microscopy

The APE1–DNA complexes were deposited on functionalized mica and functionalized with 1-(3-aminopropyl)-silatrane, as described previously [29]. Briefly, 3–4 μL of APE1-DNA reaction mixture was deposited on the functionalized mica surface, incubated for 2 min, rinsed with deionized water, dried with argon, and stored under vacuum until imaged.

A typical AFM image scanned 3 × 3 μm area with 1536 pixels/line under ambient conditions. Imaging was performed with a MultiMode 8 AFM system using TESPA probes (Bruker Nano, Camarillo, CA, USA).

### 4.5. Data Analysis

The contour length of the bare DNA, the APE1–DNA complexes, and the looped APE1–DNA complexes were measured using FemtoScan software (version 2.4.10, Advanced Technologies Center, Moscow, Russia) as described previously [29], which allows reliable tracing of DNA, as shown in Figure S1. Figures S2 and S3 illustrate the measurements of the protein position and loop size, respectively.

The yield of complexes was calculated by comparing the number of free DNA molecules with DNA molecules with APE1. The yield of looped complexes was also calculated based on the comparison with free DNA, and not the APE1–DNA complexes, and provides an absolute yield percentage.

### 4.6. Height and Volume of APE1

Grain analysis (FemtoScan software) was performed to measure the height and volume of the free APE1, APE1 complexed with DNA, and APE1 in looped complexes.

## 5. Conclusions

Our data shed light on whether APE1 recognizes G-rich regions [43]. AFM images of the DNA templates (Figure 1) demonstrate that the G-rich DNA segments were smooth and indistinguishable from the control. In contrast, G quadruplexes were considerably wider than the DNA duplex and could be visualized routinely with AFM [38,39]. Thus, no quadruplexes were formed stably in the G-rich DNA substrate. At the same time, the specificity of APE1 to G-rich sequences was shown, with approximately two-fold yield compared to non-G construct, suggesting that APE1 can bind to G-rich sequences without converting them into G quartet [14,44]. Our studies demonstrate that monomeric APE1 is capable of bridging two distant DNA segments, suggesting that APE1 should have two DNA binding sites. We hypothesize that one of them is specific for binding to G-rich sequences, and based on this hypothesis, we proposed the site search mechanism for APE1.

## Figures and Tables

**Table 1:** The yield of APE1–DNA complexes formed on G-rich and non-G-rich substrates.

Substrate	N = 500	APE1–DNA Complex (Non-Looped Complexes)	APE1–DNA Complex (Complexes with Loops)
G rich-DNA	Yield [%].	31%	22%
Non-G rich DNA	Yield [%].	15%	4%

## Data Availability

All data are included in the manuscript and the Supplementary Materials.

## Supplementary Materials

The following supporting information can be downloaded at: https://www.mdpi.com/article/10.3390/ijms25105145/s1, A detailed description of the DNA contour length, APE-1 positioning, and loop complexes measurements was provided.

## References

[1] Roychoudhury S., Pramanik S., Harris H.L., Tarpley M., Sarkar A., Spagnol G., Sorgen P.L., Chowdhury D., Band V., Klinkebiel D. (2020). Endogenous oxidized DNA bases and APE1 regulate the formation of G-quadruplex structures in the genome. Proc. Natl. Acad. Sci. USA.

[2] Pramanik S., Chen Y., Song H., Khutsishvili I., A Marky L., Ray S., Natarajan A., Singh P.K., Bhakat K.K. (2022). The human AP-endonuclease 1 (APE1) is a DNA G-quadruplex structure binding protein and regulatesKRASexpression in pancreatic ductal adenocarcinoma cells. Nucleic Acids Res..

[3] Li M., Wilson D.M. (2014). Human Apurinic/Apyrimidinic Endonuclease 1. Antioxid. Redox Signal..

[4] Bhakat K.K., Izumi T., Yang S., Hazra T.K., Mitra S. (2003). Role of acetylated human AP-endonuclease (APE1/Ref-1) in regulation of the parathyroid hormone gene. EMBO J..

[5] Thakur S., Sarkar B., Cholia R.P., Gautam N., Dhiman M., Mantha A.K. (2014). APE1/Ref-1 as an emerging therapeutic target for various human diseases: Phytochemical modulation of its functions. Exp. Mol. Med..

[6] Tell G., Quadrifoglio F., Tiribelli C., Kelley M.R. (2009). The Many Functions of APE1/Ref-1: Not Only a DNA Repair Enzyme. Antioxid. Redox Signal..

[7] Thakur S., Dhiman M., Tell G., Mantha A.K. (2015). A review on protein–protein interaction network of APE1/Ref-1 and its associated biological functions. Cell Biochem. Funct..

[8] Evans A.R., Limp-Foster M., Kelley M.R. (2000). Going APE over ref-1. Mutat. Res. Repair.

[9] Frossi B., Antoniali G., Yu K., Akhtar N., Kaplan M.H., Kelley M.R., Tell G., Pucillo C.E. (2019). Endonuclease and redox activities of human apurinic/apyrimidinic endonuclease 1 have distinctive and essential functions in IgA class switch recombination. J. Biol. Chem..

[10] Oliveira T.T., Coutinho L.G., de Oliveira L.O.A., Timoteo A.R.d.S., Farias G.C., Agnez-Lima L.F. (2022). APE1/Ref-1 Role in Inflammation and Immune Response. Front. Immunol..

[11] Hoitsma N.M., Norris J., Khoang T.H., Kaushik V., Chadda R., Antony E., Hedglin M., Freudenthal B.D. (2023). Mechanistic insight into AP-endonuclease 1 cleavage of abasic sites at stalled replication fork mimics. Nucleic Acids Res..

[12] Jaiswal A.S., Williamson E.A., Srinivasan G., Kong K., Lomelino C.L., McKenna R., Walter C., Sung P., Narayan S., Hromas R. (2019). The splicing component ISY1 regulates APE1 in base excision repair. DNA Repair.

[13] Park S.H., Kim Y., Ra J.S., Wie M.W., Kang M.-S., Kang S., Myung K., Lee K.-Y. (2021). Timely termination of repair DNA synthesis by ATAD5 is important in oxidative DNA damage-induced single-strand break repair. Nucleic Acids Res..

[14] Fleming A.M., Manage S.A.H., Burrows C.J. (2021). Binding of AP Endonuclease-1 to G-Quadruplex DNA Depends on the N-Terminal Domain, Mg^2+^, and Ionic Strength. ACS Bio Med Chem Au.

[15] Dumas L., Herviou P., Dassi E., Cammas A., Millevoi S. (2020). G-Quadruplexes in RNA Biology: Recent Advances and Future Directions. Trends Biochem. Sci..

[16] Pavlova A.V., Kubareva E.A., Monakhova M.V., Zvereva M.I., Dolinnaya N.G. (2021). Impact of G-Quadruplexes on the Regulation of Genome Integrity, DNA Damage and Repair. Biomolecules.

[17] Miclot T., Corbier C., Terenzi A., Hognon C., Grandemange S., Barone G., Monari A. (2021). Forever Young: Structural Stability of Telomeric Guanine Quadruplexes in the Presence of Oxidative DNA Lesions. Chem.–A Eur. J..

[18] Vascotto C., Fantini D., Romanello M., Cesaratto L., Deganuto M., Leonardi A., Radicella J.P., Kelley M.R., D’Ambrosio C., Scaloni A. (2009). APE1/Ref-1 Interacts with NPM1 within Nucleoli and Plays a Role in the rRNA Quality Control Process. Mol. Cell. Biol..

[19] Curtis C.D., Thorngren D.L., Ziegler Y.S., Sarkeshik A., Yates J.R., Nardulli A.M. (2009). Apurinic/Apyrimidinic Endonuclease 1 Alters Estrogen Receptor Activity and Estrogen-Responsive Gene Expression. Mol. Endocrinol..

[20] Lee Y.R., Kim K.M., Jeon B.H., Choi S. (2015). Extracellularly secreted APE1/Ref-1 triggers apoptosis in triple-negative breast cancer cells via RAGE binding, which is mediated through acetylation. Oncotarget.

[21] Woo J., Park H., Sung S.H., Moon B.-I., Suh H., Lim W. (2014). Prognostic Value of Human Apurinic/Apyrimidinic Endonuclease 1 (APE1) Expression in Breast Cancer. PLoS ONE.

[22] Bhakat K.K., Mantha A.K., Mitra S. (2009). Transcriptional Regulatory Functions of Mammalian AP-Endonuclease (APE1/Ref-1), an Essential Multifunctional Protein. Antioxid. Redox Signal..

[23] Matthews K.S. (1992). DNA looping. Microbiol. Rev..

[24] Cournac A., Plumbridge J. (2013). DNA Looping in Prokaryotes: Experimental and Theoretical Approaches. J. Bacteriol..

[25] Perez P.J., Clauvelin N., Grosner M.A., Colasanti A.V., Olson W.K. (2014). What Controls DNA Looping?. Int. J. Mol. Sci..

[26] Fogg J.M., Judge A.K., Stricker E., Chan H.L., Zechiedrich L. (2021). Supercoiling and looping promote DNA base accessibility and coordination among distant sites. Nat. Commun..

[27] Vilar J.M., Saiz L. (2005). DNA looping in gene regulation: From the assembly of macromolecular complexes to the control of transcriptional noise. Curr. Opin. Genet. Dev..

[28] Lyubchenko Y.L. (2018). Direct AFM visualization of the nanoscale dynamics of biomolecular complexes. J. Phys. D Appl. Phys..

[29] Vemulapalli S., Hashemi M., Lyubchenko Y.L. (2021). Site-Search Process for Synaptic Protein-DNA Complexes. Int. J. Mol. Sci..

[30] Shlyakhtenko L.S., Gilmore J., Portillo A., Tamulaitis G., Siksnys V., Lyubchenko Y.L. (2007). Direct visualization of the EcoRII− DNA triple synaptic complex by atomic force microscopy. Biochemistry.

[31] Lushnikov A.Y., Potaman V.N., Oussatcheva E.A., Sinden R.R., Lyubchenko Y.L. (2006). DNA Strand Arrangement within the SfiI-DNA Complex: Atomic Force Microscopy Analysis. Biochemistry.

[32] Pan Y., Shlyakhtenko L.S., Lyubchenko Y.L. (2020). High-speed atomic force microscopy directly visualizes conformational dynamics of the HIV Vif protein in complex with three host proteins. J. Biol. Chem..

[33] Li X., Sánchez-Ferrer A., Bagnani M., Adamcik J., Azzari P., Hao J., Song A., Liu H., Mezzenga R. (2020). Metal ions confinement defines the architecture of G-quartet, G-quadruplex fibrils and their assembly into nematic tactoids. Proc. Natl. Acad. Sci. USA.

[34] Zhou W., Lai R., Cheng Y., Bao Y., Miao W., Cao X., Jia G., Li G., Li C. (2023). Insights into How NH_4_^+^ Ions Enhance the Activity of Dimeric G-Quadruplex/Hemin DNAzyme. ACS Catal..

[35] Tong X., Ga L., Eerdun C., Zhao R., Ai J. (2022). Simple Monovalent Metal Ion Logical Order to Regulate the Secondary Conformation of G-Quadruplex. ACS Omega.

[36] Schultze P., Hud N.V., Smith F.W., Feigon J. (1999). The effect of sodium, potassium and ammonium ions on the conformation of the dimeric quadruplex formed by the Oxytricha nova telomere repeat oligonucleotide d(G4T4G4). Nucleic Acids Res..

[37] Marathias V.M., Bolton P.H. (1999). Determinants of DNA Quadruplex Structural Type: Sequence and Potassium Binding. Biochemistry.

[38] Neaves K.J., Huppert J.L., Henderson R.M., Edwardson J.M. (2009). Direct visualization of G-quadruplexes in DNA using atomic force microscopy. Nucleic Acids Res..

[39] Abdelhady H.G., Allen S., Davies M.C., Roberts C.J., Tendler S.J.B., Williams P.M. (2003). Direct real-time molecular scale visualisation of the degradation of condensed DNA complexes exposed to DNase I. Nucleic Acids Res..

[40] Kladova O.A., Bazlekowa-Karaban M., Baconnais S., Piétrement O., Ishchenko A.A., Matkarimov B.T., Iakovlev D.A., Vasenko A., Fedorova O.S., Le Cam E. (2018). The role of the N-terminal domain of human apurinic/apyrimidinic endonuclease 1, APE1, in DNA glycosylase stimulation. DNA Repair.

[41] Pugacheva E.M., Kubo N., Loukinov D., Tajmul M., Kang S., Kovalchuk A.L., Strunnikov A.V., Zentner G.E., Ren B., Lobanenkov V.V. (2020). CTCF mediates chromatin looping via N-terminal domain-dependent cohesin retention. Proc. Natl. Acad. Sci. USA.

[42] Mantha A.K., Oezguen N., Bhakat K.K., Izumi T., Braun W., Mitra S. (2008). Unusual Role of a Cysteine Residue in Substrate Binding and Activity of Human AP-Endonuclease 1. J. Mol. Biol..

[43] Manage S.A.H., Zhu J., Fleming A.M., Burrows C.J. (2023). Promoters vs. telomeres: AP-endonuclease 1 interactions with abasic sites in G-quadruplex folds depend on topology. RSC Chem. Biol..

[44] Reshetnikov R.V., Kopylov A.M., Golovin A.V. (2010). Classification of G-Quadruplex DNA on the Basis of the Quadruplex Twist Angle and Planarity of G-Quartets. Acta Nat..

